# Electrochemical Characterization of Charged Membranes from Different Materials and Structures via Membrane Potential Analysis

**DOI:** 10.3390/membranes13080739

**Published:** 2023-08-17

**Authors:** Virginia Romero, Lourdes Gelde, Juana Benavente

**Affiliations:** Departamento de Física Aplicada I, Facultad de Ciencia, Universidad de Málaga, 29071 Málaga, Spain; virgirom@uma.es (V.R.); geldelourdes@hotmail.com (L.G.)

**Keywords:** charged membranes, membrane potential, effective fixed charge concentration, permselectivity

## Abstract

Electrochemical characterization of positively and negatively charged membranes is performed by analyzing membrane potential values on the basis of the Teorell–Meyer–Sievers (TMS) model. This analysis allows the separate estimation of Donnan (interfacial effects) and diffusion (differences in ions transport through the membrane) contributions, and it permits the evaluation of the membrane’s effective fixed charge concentration and the transport number of the ions in the membrane. Typical ion-exchange commercial membranes (AMX, Ionics or Nafion) are analyzed, though other experimental and commercial membranes, which are derived from different materials and have diverse structures (dense, swollen or nanoporous structures), are also considered. Moreover, for some membranes, changes associated with different modifications and other effects (concentration gradient or level, solution stirring, etc.) are also analyzed.

## 1. Introduction

The presence of fixed charges on the surfaces and bulk structures of membranes used in traditional filtration separation processes (Microfiltration, Ultrafiltration, Nanofiltration and Reverse Osmosis) is a significant factor, since it affects the transport of electrolyte solutions and ions/charged particles [1,2,3]. Moreover, nowadays, charged membranes have great importance in other applications, such as fuel cells, electrodialysis, ions/organic compound recovery, power generation, food industry, microfluids (nanoporous membranes), analytical and biochemical sensors, etc., as well as models used to understand biological membranes behavior [4,5,6,7,8,9,10,11,12,13,14,15,16,17,18]. 

The presence of charges in a membrane can be associated with existing radicals or charged groups in the membrane matrix, and it could even be acquired after contact with a polar medium. Different kinds of measurements, such as streaming and membrane potentials or impedance spectroscopy, give direct and quantitative information of interest regarding the electrical/electrochemical characteristics of charged membranes, though they can also provide indirect information regarding the membrane’s structure, as well as on surface and/or bulk phase modifications [19,20,21,22]: (i) Streaming potential is associated with the movement of ions near a solid surface caused by a pressure difference [1], and it allows the characterization of the electrical double layer at the solution–membrane interface to give information regarding the membrane surface charge and other related thermodynamic parameters [23]; the solid surface can be the membrane’s surface in the case of dense membranes (tangential streaming potential) or the pore wall (transmembrane streaming potential) when porous membranes are analyzed. (ii) Membrane potential is the equilibrium electrical potential difference between two electrolyte solutions of different concentrations (but the same electrolyte) placed at both sides of a membrane, and it gives information regarding the effective fixed charge concentration present in the membrane and the transport of ions across the membrane [2,24]. (iii) Impedance spectroscopy (IS) is used for the determination of the electrical resistance and capacitance of membranes (in both dry and wet conditions, as well as in contact with electrolyte solutions or electrochemical impedance spectroscopy (EIS) using equivalent circuits as models [25,26]), which allows the determination of two basic physical properties, such as conductivity, which is a fundamental parameter required for membrane application in fuel cell, and the dielectric constant (assuming homogeneous membranes/layers with known thickness); the latter parameter was initially used for thickness estimation of the thin compact active layer of reverse osmosis membranes [27]. Moreover, these three electrochemical characterization techniques might also provide information regarding electrolyte/membrane interface and/or membrane changes associated with membrane fouling [28,29], which is one of the main problems affecting membrane applications in the different filtration processes, while membrane potential and impedance spectroscopy measurements have allowed us to perform the electrochemical characterization of hydrogels and vegetal membranes (plant cuticular membrane) [30,31]. 

In this work, electrochemical characterization of different types of positively and negatively charged commercial and experimental membranes was performed by analyzing membrane potentials (ΔΦ_mbr_) values using the Teorell–Mayer–Sievers (TMS) model [32,33], which allows the determination of the effective membrane fixed charge concentration and the ion (anion or cation) transport numbers in the membranes, as well as allowing separate estimation of two contributions, namely diffusive and interfacial or Donnan (which can not be individually measured), associated with differences in the transport/mobility of ions (mainly counter-ions) across the membranes and co-ion exclusion, respectively. Typical commercial cross-linked polyelectrolyte ion-exchange membranes for desalting purposes and a perfluorinated membrane (thermoplastic polymer with pedant sulphonic groups) for fuel cell application were selected to perform electrochemical characterization, as were an experimental polymeric inclusion membrane (PIM), which has application in the separation of heavy metals and organic compounds, and other polymeric membranes with different hydrophilicities and swelling degrees, which were made of regenerated cellulose (RC)—the most common biopolymer in nature—chitosan or exopolysaccharide (possible use in biomedicine and agricultural). An inorganic membrane, which is a nanoporous alumina membrane obtained via the aluminum anodization process, was also used, which presents a practically ideal nanoporous structure with particular optical characteristics and sensing application [34,35]. Some of these membranes were treated with an acidic solution (0.1 M H_2_SO_4_) or differently modified (inclusion of the ionic liquid cation DTA^+^ or silver nanoparticles) to see how such modifications could affect their electrochemical parameters. Moreover, the influence of external conditions (concentration level or solution stirring) were considered for some of the samples. These results show the high permselectivity of commercial cross-linked polyelectrolytes (≤90%) due to the high Donnan potential contribution for almost the whole range of concentrations studied (10^−3^–10^−1^ M NaCl), although slightly lower values were obtained in the case of the perfluorinated membrane (~85%). On the other hand, the increase in membrane material hydrophilicity or pore radius (for nanoporous samples) clearly increases the contribution of diffusion potential to ΔΦ_mbr_ values at concentrations higher than 0.02 M NaCl, reducing membrane permselectivity.

## 2. Theoretical Background

**Figure 1:**
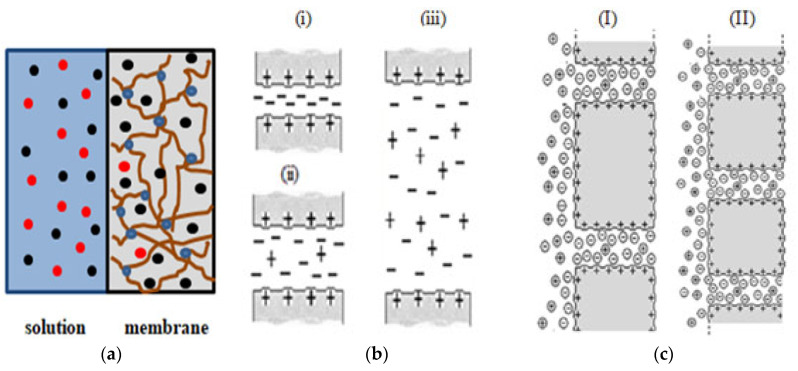
Scheme of the following aspects: (**a**) swollen membrane/solution interface: (●) fixed charge, (●) counter ions and (●) co-ions; (**b**) effect of pore size on ions transport for membranes with similar values of fixed charge: (**i**) total co-ions exclusion, (**ii**) medium co-ions exclusion, and (**iii**) low co-ions exclusion; (**c**) membranes with similar charges and pore sizes but different porosities: (**I**) lower porosity and (**II**) higher porosity.

Charged membranes in contact with electrolyte solutions usually consist of a polymeric matrix with fixed ionic groups and mobile counter-ions (ions with opposite charges to that of the membrane, for compensation reason), and sorbed electrolyte. In particular, the transport of ions through membranes separating two electrolyte solutions of different concentrations involves their inclusion in the membrane structure, starting from the high concentration solution, and their transport across the membrane to the low concentration (receiving) solution. The presence of fixed charges in the membrane surfaces and/or structures (ionizable groups bound to the membrane) provides their electropositive/electronegative characters, which significantly affect both ion inclusion and transport. In fact, the presence of positive fixed charges in the membrane provokes the exclusion of cations (membrane co-ions) and favors the inclusion of anions (membrane counter-ions), generating an electrical potential difference at each solution/membrane interface (Donnan or interfacial potential), though due to the concentration gradient in the membrane, ions move from high to low concentration solutions, generating a diffusion potential in the membrane [2]. Consequently, membrane charge and the mobility (ui) of ions in the membrane are two significant parameters related to both the solution/membrane interface interactions (hydrophobic/hydrophilic or charged/uncharged character of membranes) and the membrane material and structure (dense, swollen or nanoporous membranes). Figure 1 shows a scheme of the effect of membrane charge and structure on the solution/membrane interface and transport of ions, in which the solvent (water generally) is not indicated. As can be observed, for a given material (similar number of fixed charge), pore size significantly affects the transport of ions (Figure 1b), though porosity might also influence the solution/membrane charge distribution (Figure 1c).

With respect to the transport of ions through the membrane, taking into account electrochemical relationships [36], ionic mobility can be related to ions transport numbers, ti, which represent the fraction of the total current transported for each ion, that is, t_i_ = I_i_/I_T_ and, consequently, ∑ti = 1. Then, for single electrolyte solutions, the transport number through ideal anion exchangers (positively charged membranes with total exclusion of cations) is t_−_ = 1, while t_+_ = 1 in the case of ideal cation exchangers (negatively charged membranes with total exclusions of anions); when membrane co-ions are not totally excluded, the transport number of the counter ion in the membrane presents lower values. The determination of ion transport numbers in the membrane allows us to estimate the membrane’s permselectivity, which is a significant characteristic of membranes used in water treatment or the characterization of biological membranes [18]. Expressions of anionic/cationic permselectivity, P(−)/P(+), are as follows [2]:

(1) $$\mathrm{anionic}\;\mathrm{permselectivity}:\;P(-)=(t_{-}-t_{-}^{{}^{\circ}}\;)/t_{+}^{{}^{\circ}}$$

(2) $$\mathrm{cationic}\;\mathrm{permselectivity}:\;P(+)=(t_{+}-t_{+}^{{}^{\circ}})/t_{-}^{{}^{\circ}}$$

where t_−_/t_+_ indicate the anion/cation transport number in the membrane, and $t_{-}^{{}^{\circ}}$/$t_{+}^{{}^{\circ}}$ correspond to the anion/cation transport number in the solution. Consequently, permselectivity values do not only depend on membrane characteristics (charge and structure), but also the electrolyte solution. 

**Figure 2:**
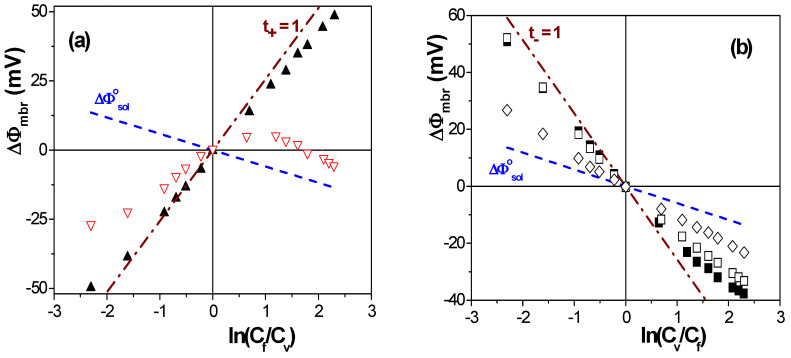
Membrane potential as a function of the solutions concentration ratio for the following samples: (**a**) Nafion-117 membrane (▲) and RC–CE membrane (∇); (**b**) nanoporous alumina membranes ALM-1 (■), ALM-2 (□) and ALM-3 (◊). Theoretical values of an ideal cation/anion exchanger (dashed-dot line) and NaCl solution diffusion (dashed line) potentials.

As an example of the influence of both membrane material and structure on interfacial effects and the transport of ions through charged membranes, the dependence of membrane potential (ΔΦ_mbr_) values on the concentration ratio of two NaCl solutions (C_v_/C_f_, with C_f_ = 0.01 M and 10^−3^ M ≤ C_v_ ≤ 0.1 M) placed at each side of different symmetric membranes, is presented in Figure 2; for comparison, theoretical values of ideal cation/anion exchangers (t_+_/t_−_ = 1), as well as for the solution (NaCl) diffusion potentials (ΔΦ^o^_dif_), are also indicated in Figure 2. Independently of the model used for membrane potential analysis, differences in the ΔΦ_mbr_ − ln(C_v_/C_f_) dependence give qualitative information of interest regarding the effect of the structure/materials on electrolyte transport. The values shown in Figure 2a correspond to two non-porous polymeric negatively charged membranes fabricated from different materials (Nafion (hydrophobic) and regenerated cellulose or RC (hydrophilic)), denominated Nafion-117 and RC–CE, respectively. The effect of membranes’ geometrical parameters (pore size and porosity) on ΔΦ_mbr_ values can be observed in Figure 2b, where ΔΦ_mbr_ values for three nanoporous alumina membranes obtained by the aluminum anodization process [37] (with almost ideal porous structure), that is, membranes from the same material but different pore radii and porosities, are indicated (ALM-1: r_p_ = 12 nm, Θ = 12%; ALM-2: r_p_ = 23 nm, Θ = 19%; and ALM-3: r_p_ = 82 nm Θ = 10%, average values [38]). 

Results in Figure 2a show an almost ideal cation-exchange character of the Nafion-117 sample (which is commonly used in fuel cell applications) with values very similar to those of ideal cation-exchangers, while the ΔΦ_mbr_ values determined for the RC–CE membrane indicate a lower effect of the negative fixed charges (associated with the oxidation of –CH_2_OH groups to –COOH [39]), even at low NaCl concentrations; the membrane fixed-charges seem to be practically masked/neutralized at solution concentrations higher than 0.02 M NaCl, changing the tendency of ΔΦ_mbr_ values to that shown by the NaCl solution diffusion potential, which is attributed to electrolyte inclusion in the membrane structure due to the high hydrophilic character and swelling degree of the RC–CE membrane (120% water uptake). The electropositive character of the alumina membranes and the effect of their structure on ΔΦ_mbr_ values can clearly be observed in Figure 2b: at low solution concentrations (<0.03 M NaCl), both ALM-1 and ALM-2 nanoporous membranes show values rather similar to those exhibited by an ideal anion-exchange membrane, which are attributed to cation exclusion from the positively charged surfaces of membranes, though that exclusion effect seems to be reduced at higher electrolyte concentrations due to partial charge shielding at the solution/membrane interface, which allows the increase in cations into the membrane pores. As expected, the increases in the pore radii clearly favor the contribution of cations during the transport through the membranes, ΔΦ_mbr_ values follow a tendency more similar to that of NaCl solution diffusion potential, and practically no interfacial interactions seem to exit in the case of the ALM-3 membrane. These examples clearly show the need to consider both solution/membrane interfacial effects and membrane structure in the analysis of membrane potential values, as well as the expressions able to quantify membrane charge and ion transport values related to membrane particular characteristics. 

The model proposed by the Teorell–Meyer–Sievers (TMS) model [32,33] for the analysis of membrane potential (ΔΦmbr), as well as the equilibrium potential difference between two electrolyte solutions of different concentrations separated by a membrane, considers ΔΦmbr values to be the sum of two different terms: (i) the Donnan or interfacial potential (ΔΦDon), which is related to the exclusion of co-ions (ions with the same sign that the membrane charge) due to electrical repulsion, and (ii) the diffusion potential (ΔΦdif), which is associated with the concentration gradient caused by the different mobility of ions (or transport number) through the membrane. Then, an expression of membrane potential is obtained through the addition of both effects, that is, ΔΦmbr = ∆øDon(I) + ∆ødif + ∆øDon(II), where I and II indicate each membrane/solution interface. 

The following expressions of diffusion and Donnan potentials are considered to assume no pressure gradient and isothermal conditions, while concentrations instead of activities are used for reasons of simplicity [2]:

(i) Diffusion potential:
  (3) $$∆\Phi_{dif}=-\frac{\mathrm{RT}}{wzF}\left[U\ln\frac{\sqrt{4y_{v}^{2}+1}\;+wU}{\sqrt{4y_{f}^{2}+1}\;+wU}\right]$$
  where w is −1/+1 for negatively/positively charged membranes; z_i_ is the ion valency; X_ef_ is the effective concentration of fixed charges in the membrane; R and F are the gas and Faraday constants, respectively; and T is the temperature of the system. y_i_ = K_s,i_C_i_/│X_ef_│, with i = v/f (solution concentration values at each side of the membrane), and K_s,i_ is the partition coefficient and membrane/solution concentration ratio of K_+,i_ = $\bar{C_{+,i}}$/$C_{i}$ and K_−,i_ = $\bar{C_{+,i}}$/$C_{i}$, where the upper bar refers to the concentration inside of the membrane, with K_s,i_ = 1 in the case of porous or highly hydrophilic (or swelling) membranes, while membrane electroneutrality condition establishes a relationship between these parameters: $w\left|X_{ef}\right|+\bar{C_{+}}+\bar{C_{-}}$ = 0 [2]. Parameter U is related to ion diffusion coefficient D_i_ and ion transport numbers [2]: U = ([t_+_/│z_+_│] − [_t−_/│z│]), then for 1:1 electrolytes, U = (t_+_ − t_−_) = (2t^+^ − 1). Electrolyte solution diffusion potentials (∆ø^o^dif) could be obtained from Equation (3), eliminating all membrane contribution, that is, ∆ø^o^dif = (RT/F)[(t^o^_+_/│z_+_│) − ($t_{-}^{{}^{\circ}}$/│z_−_│)]ln(C_1_/C_2_), and for 1:1 electrolytes: ∆ø^o^dif = (RT/F)[(1 − 2$t_{+}^{{}^{\circ}}$)]ln(C_1_/C_2_).
(ii) Donnan potential [2]:
  (4) $$∆\Phi_{Don}=-\frac{\mathrm{RT}}{wzF}\ln\left[\frac{C_{f}}{C_{v}}\frac{\sqrt{4y_{v}^{2}+1}\;+w}{\sqrt{4y_{f}^{2}+1}\;+w}\right]$$

In this context, we should indicate the impossibility of measuring, via electrochemical techniques, the individual components of membrane potential; consequently, direct measurements of Donnan potential cannot be performed. However, recently, the use of the tender ambient pressure X-ray photoelectron spectroscopy (AP-XPS) method, which is based on the binding energy shift of the membrane equilibrated with salt solutions, has been proposed for use in measuring Donnan potential [40]. 

Then, according to the TMS model, the membrane potential can be expressed as follows:

(5) $$∆\Phi_{mbr}=-\frac{\mathrm{RT}}{wzF}\left[U\ln\frac{\sqrt{4y_{v}^{2}+1}\;+wU}{\sqrt{4y_{f}^{2}+1}\;+wU}-\ln\frac{Cf}{Cv}\frac{\sqrt{4y_{v}^{2}+1}\;+w}{\sqrt{4y_{f}^{2}+1}\;+w}\right]$$

On the other hand, different effects (steric/frictional or dielectric) could influence the inclusion/transport of electrolyte solutions for membranes with very narrow pores (such as those used for the nanofiltration process, pore radii ≤1 nm), and, consequently, Equation (5) has to be modified to include particular factors related to those effects [41,42].

The fit of the data points shown in Figure 2a to Equation (5) allows us to determine the following values of the effective fixed charge concentrations and the ion transport number trough for the Nafion-117 and the RC–CE membranes: X_ef_^Nafion−117^ = −0.23 M and X_ef_^RC–CE^ = −0.016 M, as well as t_+_^Nafion−117^ = 0.90 and t_+_^RC–CE^ = 0.69. For the alumina membranes, the values obtained are as follows: X_ef_^ALM−1^ = + 0.012 M, X_ef_^ALM−2^ = + 0.008 M and X_ef_^ALM−3^ = + 0.003 M, while t_−_^ALM−1^ = 0.74, t_−_^ALM−2^ = 0.72 and t_−_^ALM−3^ = 0.657; the latter value does not differ significantly (6%) from that determined when only considering diffusion potential (without any solution/membrane interfacial or Donnan contribution) due to the high pore radii of the ALM-3 membrane, even for a sample with a relatively compact structure (10% porosity). Permselectivity values of these membranes were determined by using Equations (1) and (2), and the results are as follows: P(−)^ALM−1^ = 32.5%, P(−)^ALM−2^ = 27.3%, P(−)^ALM−3^ = 11.0%, P(+)^Nafion−117^ = 83.7% and P(+)^RC–CE^ = 49.6%. 

Other factors, such as concentration level, solution stirring rate or the type of electrolyte solution, might affect membrane potential values, and comparisons between these effects (stirred/non-stirred solutions for C_f_ = 10^−2^ M), NaCl concentrations levels (C_f_ = 10^−3^ M and 10^−2^ M) or the electrolyte solutions (C_f_ = 10^−2^ M NaCl and 10^−2^ M KCl) for the ALM-2 membrane are presented in the Supplementary Information document (Figure S1). As can be observed in the figure, the increase in the concentration level seems to slightly decrease the interfacial exclusion of cations, enlarging their contribution to the transport of charges, while an opposite effect is obtained for non-stirring solutions values, which is attributed to a concentration increase near the membrane’s surface. The reduction in ΔΦ_mbr_ values of the KCl solution at high concentrations, which is dominated by diffusion contribution, is associated with the higher transport number of K^+^ than Na^+^. On the other hand, differences in membrane potential values due to the use of concentrations instead of activities are also points of interest, and a comparison between the results obtained for Nafion-117 and ALM-1 membranes is shown in the Supplementary Information document (Figure S2), in which very similar values can be observed; in fact, the fit of these data points gives the following values of the effective fixed charge concentration and cation transport number for the Nafion-117 membrane: X_ef_^Nafion−117^ (actv)= −0.233 M and t_+_^Nafion−117^ (actv) = 0.886 (change ~1.5% with respect to concentration values), which have fit errors of 4.6%, while practically negligible differences in both fitted values were determined for the ALM-1 membrane. 

It should be indicated that different approximations, which are usually valid for weekly charged membranes, have been proposed for the estimation of X_ef_ and t_i_ values. For instance, when the ΔΦ_mbr_ – ln(C_v_/C_f_) dependence presents two branches (as membrane RC–CE), an expression considers the following issues: (i) the value of the variable concentration in the maximum of the curve (C_v-ext_); and (ii) the slope of branch-curve side corresponding to high concentrations (U), which was proposed for the estimation of the membrane’s fixed charge concentration [43], X_ef_ = 2C_v-ext_U/(1 − U^2^)^1/2^ (for 1:1 electrolytes). This analysis provides the following values of the membrane fixed charge concentration and cation transport number for the RC–CE membrane: X_ef_^RC–CE^ = −0.016 M and t_+_^RC–CE^ = 0.669, that is, a similar X_ef_ value and only a difference of 4% in the transport number of the cation. 

On the other hand, as was previously mentioned, the presence of charges in the membrane matrix affects the values of other characteristic parameters of membranes, such as the permeation/diffusion coefficients (P_s_/D_s_), and different expressions that allow the estimation of values of the membrane fixed charge concentration by analyzing P_s_/D_s_ versus feed concentration (measurements were also performed under a concentration gradient) have been proposed [44,45]. In particular, variations in solution permeability with feed concentrations for RC–CE and ALM-1 membranes are presented in the Supplementary Information document (Figure S3), and the following values of │X_ef_│ and D_s_ were estimated using the Filippov et al. model [44]: │X_ef_│^ALM−1^ = 0.013 M and D_s_^ALM−1^ = 3.6 × 10^−10^ m^2^/s, as well as │X_ef_│^RC–CE^ = 0.015 M and D_s_^RC–CE^ = 1.3 × 10^−10^ m^2^/s. As can be observed, membrane effective fixed charge values are practically similar to those already determined using the membrane potential results, supporting the reliability of the values previously obtained by analyzing ΔΦ_mbr_ values, although no information regarding the nature of the electrical character of membranes (positively/negatively charged) is provided via permeability measurement, while the lower value of the NaCl diffusion coefficient in the membrane (around one order of magnitude) compared to the solution [36] is attributed to frictional effects. 

The analyses presented in this section demonstrate the possibility of finding qualitative and quantitative information of interest from membrane potential measurements when the transport of electrolyte solutions across different kinds of charged membranes is studied, which can be extended by considering other electrochemical experimental techniques. 

## 3. Materials and Methods

### 3.1. Materials

Commercial and experimental membranes obtainedfrom different materials, with diverse structures (dense, swollen or nanoporous), for different applications have been selected to show the electrochemical information that can be obtained by analyzing membrane potential values. The chosen membranes are as follows:

- An anion-exchange membrane, specifically an AMX-Sb sample (Astom, Tokyo, Japan), consisting of a copolymer of styrene and divinylbenzene matrix with quaternary ammonium fixed groups [19].
- Two commercial ion-exchange membranes provided by Ionics Iberica (Las Palmas de Gran Canaria, Spain); one membrane was positively charged (AR204-SZRA-412) and the other membrane was negatively charged (CR67-HMR-402). These membranes were prepared using vinyl monomer and acrylic fiber with –N^+^(CH_3_) or –SO_3_ radicals to provide them positive/negative characters [46], and they will be named hereafter as Ionics(+) and Ionics(−). To estimate possible changes in ion transport associated with membrane contact with acidic solutions, both samples were maintained for one year in a 0.a M H_2_SO_4_ solution, and they were denominated as Ionic(+)/H_2_SO_4_ and Ionic(−)/H_2_SO_4_, respectively.
- A polymer inclusion membrane (PIM) was obtained using cellulose triacetate (CTA) as base-polymer and the ionic liquid AliquatCl (tricaprylmethylammonium chloride (C_25_H_55_N^+^Cl^−^ or commercially, Aliquat 336) in proportions (in mass) of 70% CTA and 30% AliquatCl [10], and it was denominated as 70CTA/30AlqCl. This membrane was prepared by the research group of Dr. C. Fontás and Dr. E. Anticó, Analytical Chemical Department, Gerona University, Gerona, Spain.
- A symmetric nanoporous alumina membrane was obtained via the two-step anodization process [37] using 0.3 M H_2_SO_4_ solution as the electrolyte and 40 V as the anodizing potential (similar to sample ALM-1), with external and internal (pore walls) surfaces being coated with an Al_2_O_3_ layer via the atomic layer deposition (ALD) method [47]; Al_2_O_3_ coating reduced pore-radii and porosity without modification of the surface nature (r_p_ = 9 nm, Θ = 6% average values, see SEM pictures in the Supplementary Information document (Figure S4)). This modified membrane was called ALM-1/Al_2_O_3_, and it was obtained, as was the ALM-1 membrane, by Prof. V. de la Prida and Dr. V. Vega (Nanomembranes Laboratory, Oviedo University, Oviedo, Spain).
- A negatively charged commercial membrane Nafion-112 (in protonated form) from Dupont (USA). This membrane, commonly used for fuel cell application, was modified by incorporation of the ionic liquid cation n-dodecyltrimethylammonium (DTA^+^) through a proton/cation exchange process, maintaining the Nafion membrane during 24 h in a 60% aqueous solution of the IL (C_12_H_25_N(CH_3_)_3_Cl or DTACl). This membrane was denominated as Nafion-112/DTA^+^, and it shows higher chemical stability than the Nafion-112 at temperatures higher than 80 °C [48].
- Two experimental bio-based polymeric membranes—a microbial exopolysaccharide hydrophilic membrane (EPS sample, 103% swelling degree) composed of sugars (galactose (68%), glucose, mannose and rhamnose) and acyl groups (pyruvil, succynil and acetyl) [49]—and a Chitosane membrane (average contact angle: 58°) derived from chitin (the most abundant natural amino polysaccharide) [50]. These membranes were prepared at the Universidade Nova de Lisboa (Portugal) by the research groups of Prof. M.A.M. Reis (Chemistry Department) and Prof. I.M. Coelhoso (Chemical Engineering Department).
- A highly hydrophilic regenerated cellulose membrane (RC–CE sample, from Cellophane Española, Burgos, Spain) modified via inclusion of silver nanoparticles (RC–CE/AgNPs membrane). The Ag NPs were supplied by Prof. M. López-Romero (Icon Nanotech, Málaga, Spain). The RC–CE/AgNPs membrane was obtained by introducing a piece of the RC–CE membrane into an aqueous solution of Ag nanoparticles for 1 h. Ag NPs are commonly included in the structure of polymeric membranes as a way to increase mechanical stability (strain–stress curves were shown as Supplementary Information document (Figure S5)) and biofouling reduction [51].

### 3.2. Membrane Potential Determination

The equilibrium electrical potential difference (or cell potential, ΔE) was measured using Ag/AgCl electrodes (reversible to ion Cl^−^) connected to a digital voltmeter (Yokohama 7552, 1 GΩ input resistance) in a dead-end test cell with magnetic stirrers to minimize concentration–polarization at the membrane surfaces (stirring rate: 550 rpm). Most of these measurements were carried out with NaCl solutions (at 25 ± 2 °C and pH = 5.9 ± 0.2) by keeping fixed the concentration of the solution at one side of the membrane (Cf = 0.01 M) and gradually changing the concentration of the solution at the other side (0.002 M ≤ C_v_ ≤ 0.1 M). Prior to measurements, the membranes were maintained overnight in contact with the lower concentration solution. Membrane potential (ΔΦ_mbr_) values were obtained by subtracting the electrode potential (ΔΦ_elect_ = −(RT/Fz)ln(C_v_/C_f_)) from the cell potential of each pair of C_v_/C_f_ concentrations, that is, ΔΦ_mbr_ = ΔE − ΔΦ_elect_. Equilibrium ΔE values were recorded until a constant value was reached, which depends on the membranes characteristics’.

## 4. Results

**Figure 3:**
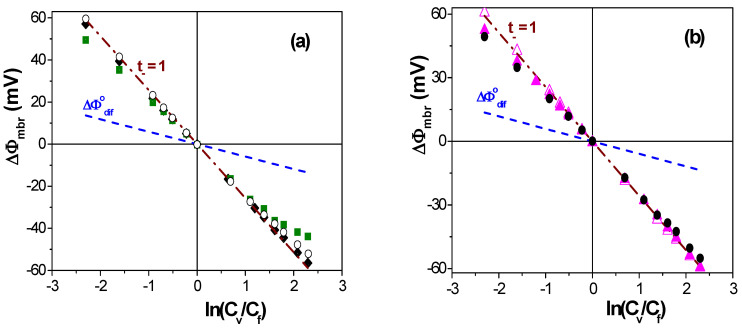
Membrane potential as a function of solution concentration ratios for the following membranes: (**a**) AMX-Sb (♦), nanoporous ALM-1/Al_2_O_3_ (o) and Chitosan (■) membranes; (**b**) Ionics(+) (Δ), Ionics(+)/H_2_SO_4_ (▲) and 70CTA/30AlqCl (●) membranes. Theoretical membrane potential values of an ideal anion-exchange membrane (dashed-dot line) and NaCl solution diffusion potential (dashed line).

**Figure 4:**
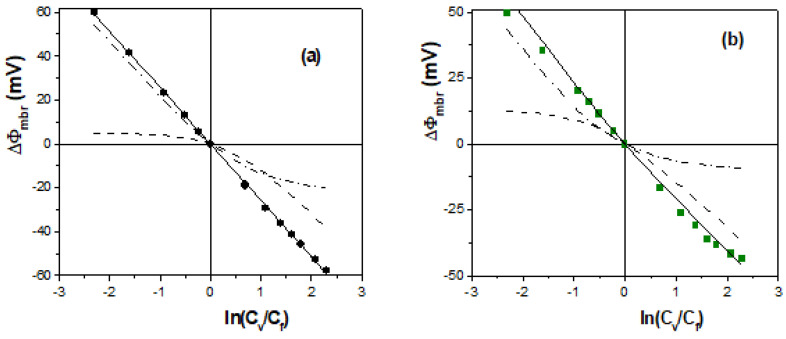

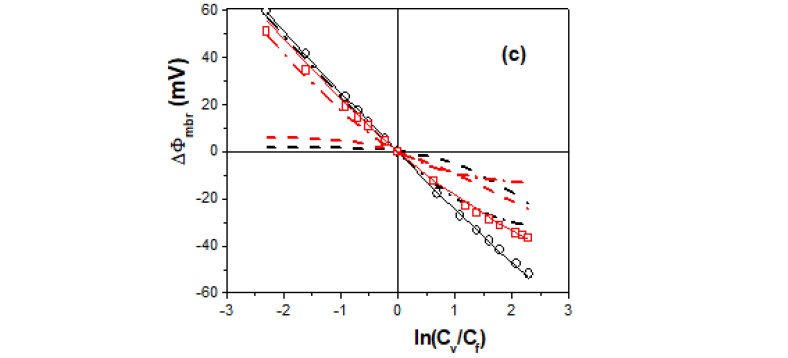
Comparison between experimental (points) and theoretical membrane potential values (solid line), as well as Donnan (dashed-dot line) and diffusion (dashed line) contributions. (**a**) 70CTA/30AlqCl membrane (●) (**b**) Chitosan membrane (■). (**c**) ALM-1/Al_2_O_3_ (o) and ALM-1 (□) membranes.

Electrochemical characterization of the studied membranes was performed by analyzing the dependence of the membrane potential on the ratio of solution concentrations at both sides of the membrane. Figure 3 shows the ΔΦ_mbr_ − ln(C_v_/C_f_) values obtained for the positively charged samples; in particular, values corresponding to membranes AMX-Sb, Chitosan and ALM-1/Al_2_O_3_ are shown in Figure 4a, while Figure 4b shows those for Ionic(+), Ionic(+)/H_2_SO_4_ and 70CTA/30AlqCl membranes. Theoretical values of an ideal positively charged membrane (t_−_ = 1) and the solution (NaCl) diffusion potential (ΔΦ_dif_^o^) are also indicated in Figure 4a,b.

Values of the effective concentration of the fixed charge in the membranes and the anion transport numbers were obtained via fitting to Equation (5) the data points shown in Figure 3, and the values and the error fit obtained are indicated in Table 1. From t_−_ values, anionic permeability for the different membranes was determined via Equation (1), and their values are indicated in Table 1. As can be observed, the ion-exchange membranes (AMX-Sb and Ionics(+)) show very high values of the anion transport number and permselectivity (almost higher that 90%), even with differences in the values of effective fixed charge, although they are significantly higher than those presented by the other membranes, though these results show the practically null effect on effective charge and anion transport number caused by one-year immersion of the Ionics(+) membrane in a 0.1 M H_2_SO_4_ solution. It should be indicated that there is not a direct correlation between the X_ef_ value and that corresponding to the ion-exchange capacity of membranes (determined via titration) usually given by suppliers, which is 1.3–1.4 meq/g dry resin for AMX-Sb and 2.1 meq/g dry resin for Ionics(+) membranes [20,52], since other materials and structural factors are involved (water content, thickness, or even charge location, since ΔΦ_mbr_ values include a diffusive process [17,52]). The 70CTA/30AlqCl polymer inclusion membrane also shows a practically anionic ideal behavior at high solution concentrations, though its values at low concentrations slightly differ from it; the Chitosan membrane shows lower absolute values of membrane potential for the whole range of concentrations, which indicate lower interfacial effects and higher co-ion transport across the membrane associated with its swelling capacity, which reduces its permselectivity. On the other hand, the material/structure effect on electrochemical parameters can be observed by comparing the results obtained for the dense 70CTA/30AlqCl membrane (around 1% solution uptake) and the nanoporous ALM-1/Al_2_O_3_ membrane, although the effective fixed charge concentration value obtained for the alumina membrane is higher than that obtained for the70CTA/30AlqCl membrane (~15%), and the anion transport number and permselectivity are lower due to solution inclusion in the alumina membrane structure (nanopores).

Figure 4 shows a comparison between experimental and theoretical values of Chitosan, 70CTA/0AlqCl and ALM-1/Al_2_O_3_ membranes, as well as the corresponding Donnan and diffusion potential contributions. These results clearly show the higher Donnan contribution exhibited by the 70CTA/30AlqCl membrane (Figure 4a) at low concentrations (around 88% of membrane potential values), maintaining an average value of—(16 ± 2) mV for concentrations higher than 0.02 M NaCl, while the Donnan contribution for Chitosan membrane (Figure 4b) ranges between ~75% of the membrane potential value at low concentrations and only 20% at the highest concentrations (>0.3 M NaCl), which is in agreement with the lower value of the effective fixed charge value and hydrophilic character. The ALM-1/Al_2_O_3_ membrane also behaves as a practically ideal anion-exchange membrane at low concentrations, since 92% of ΔΦ_mbr_ values of C_v_ < 0.06 M correspond to the Donnan contribution, before decreasing to 35% at higher concentrations. Moreover, the comparison between ΔΦ_mbr_ values of the ALM-1/Al_2_O_3_ membrane and those previously determined regarding the ALM-1 membrane (Figure 2b) shows the significant reduction in the diffusion potential contribution associated with pore-size/porosity decrease (~40% for C_v_ > 0.05 M NaCl). 

**Figure 5:**
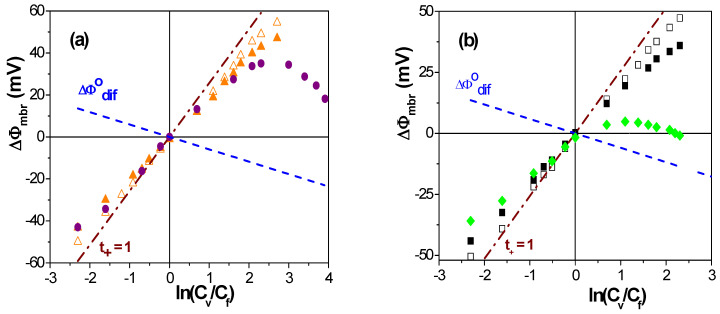
Membrane potential as a function of solution concentration ratios for the following membranes: (**a**) Ionics(−) (Δ), Ionics(−)/H_2_SO_4_ (▲) and EPS (●) membranes; (**b**) Nafion-112 (□), Nafion-112/DTA^+^ (■) and RC–CE/AgNPs membranes (♦). Theoretical membrane potential values of an ideal cation-exchange membrane (dashed-dot line) and NaCl solution diffusion potential (dashed line).

The dependence of membrane potential values on concentration ratios obtained for the negatively charged membranes is shown in Figure 5, as are those for an ideal cation-exchange membrane and the NaCl diffusion potentials, which provide qualitative information regarding membranes characteristics. As can be observed in Figure 5a, the Ionics(−) membrane shows a behavior very similar to that of an ideal cation-exchange membrane (just opposite to that obtained for the Ionics(+) one), and its immersion in a 0.1-molarity H_2_SO_4_ solution for one year does not seem to affect the ΔΦ_mbr_–ln(C_v_/C_f_) linear tendency, which only slightly reduces ΔΦ_mbr_ values (~2%); the polymeric EPS membrane also shows high cation-exchange behavior for concentrations lower than 0.08-molarity NaCl, though a clear change in the ΔΦ_mbr_–ln(C_v_/C_f_) tendency is obtained at higher concentrations (the range of C_v_ values was increased to 0.5-molarity NaCl for clear establishment of sample tendency change), which is attributed to partial neutralization of the membrane’s fixed-charge at high solution concentrations, which would favor the transport of co-ions through the structure of this hydrophilic biopolymer, as was previously shown in Section 2. This effect is clearly observed in Figure 5b for the RC–CE/AgNPs membrane due to the hydrophilic character of the regenerated cellulose and the high fractional void volume (FVV) of this membrane, although certain reduction in the FVV due to the presence of the AgNPs could be responsible for the slightly higher absolute values of the RC–CE/AgNPs membrane than for the RC–CE membrane (Figure 1a). Moreover, physicochemical surface differences between Nafion-112 and Nafion-112/DTA^+^ membranes (obtained by XPS and contact angle measurements) might explain differences in ΔΦ_mbr_ values observed in Figure 5b for these membranes, which were associated with the increase in the hydrophilic character of the Nafion-112/DTA^+^ sample (19% reduction in contact angle); in fact, the inclusion of ionic liquids cations in Nafion membranes seems to favor water content, reducing the samples’ hydrophobicity [4,53,54]. 

Results in Table 2, which were obtained via the fit to Equation (5) of the experimental values of the negatively charged membranes indicated in Figure 5, show the values determined for the effective membrane fixed charge concentration, cation transport number and cationic permselectivity (by Equation (2)), allowing the quantification of the effects described above. Fixed charge concentration and permselectivity for Ionics(−) and Ionics(−)/H_2_SO_4_ membranes are slightly higher than those exhibited by the positively charged samples (Table 1), though in this case, immersion for one year in a 0.1-molarity H_2_SO_4_ solution slightly reduces X_ef_ and permselectivity values (14% and 4%, respectively), which could be associated with changes in the polymeric fabric. X_ef_, t_+_ and P(−) values of the Nafion-112 membrane are similar to those obtained for Nafion-117 (Section 2), though its modification using the IL cation DTA^+^ reduces these figures (49%, 14% and 26%, respectively) and, more significantly, slightly varies the ΔΦ_mbr_–ln(C_v_/C_f_) linear tendency to that exhibited by hydrophilic materials, which is in concordance with the lower contact angle of the modified membrane. In this case, the higher X_ef_ value obtained for Ionics(−) membrane than for the Nafion-112 is in agreement with its higher ion-exchange capacity (2.1 meq/g dry resin [52] and 0.9 meq/g dry membrane, respectively). Change in the linear tendency with the increase in the concentration is also presented for both EPS and RC–CE/AgNPs membranes, necessarily being a relatively high value of the variable concentration (C_v_ > 0.2 M NaCl) to mask or shield the higher fixed charge on the surface of EPS membrane, though a low C_v_ value (0.04 M NaCl) is needed in the case of the RC–CE/AgNPs membrane due to its lower fixed charge, high hydrophilicity and swollen degree; the effect of both charge density and water content on salt sorption for hydrogel-type membranes has already been reported [55]. A comparison between X_ef_ and P(+) values of RC–CE/AgNPs and RC–CE membranes shows increases of 14% and 3.5%, respectively, being associated with AgNPs inclusion. On the other hand, the analysis of diffusion measurements proposed by Filippov et al. [44] (P_s_ vs. C_fd_) gives the following values of the effective membrane fixed charge and electrolyte diffusion coefficient: │X_ef_^EPS^│ = 0.18 M and D_s_^EPS^ = 7.0 × 10^−11^ m^2^/s, as well as │X_ef_^RC–CE/AgNPs^│ = 0.02 M and D_s_^RC–CE/AgNPs^ = 1.8 × 10^−10^ m^2^/s. These results show X_ef_ values in rather good agreement with the two kinds of measurements (under similar experimental conditions) and analyses performed, while D_s_ results indicate a more compact structure for the EPS membrane (lower swelling degree) than for the RC–CE/AgNPs membrane.

**Figure 6:**
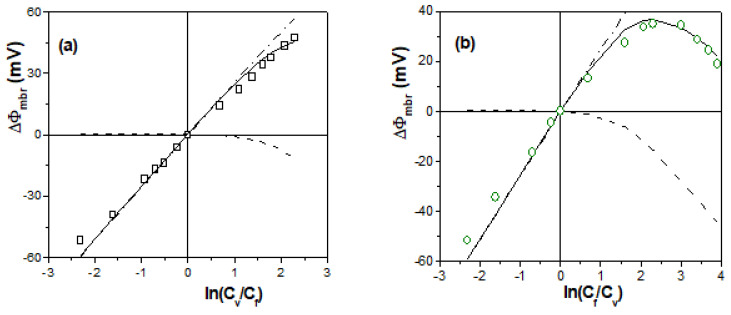
Comparison between experimental (points) and theoretical (solid line) membrane potential values, as well as Donnan (dashed-dot line) and diffusion (dashed line) contributions. (**a**) Nafion-112 membrane (□). (**b**) EPS membrane (o).

Comparison between experimental and theoretical values of Nafion-112 and EPS membranes, as well as Donnan and diffusion potential contributions, are presented in Figure 6. For both membranes, at low solution concentrations, membrane potentials practically correspond to Donnan potential, having negligible diffusion contribution, but for C_v_ > 0.05-molarity NaCl, this latter contribution increases to 10% for the Nafion-112 membrane and to 20% for the EPS membrane.

**Figure 7:**
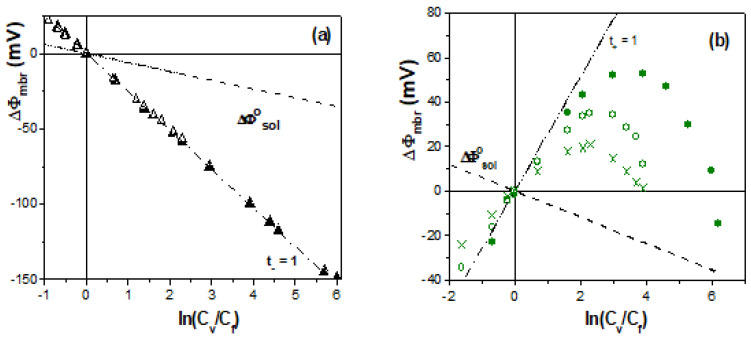
Membrane potential vales for the following membranes: (**a**) AMX membrane with stirred solutions of C_f_ = 10^−3^ M NaCl (▲) and C_f_ = 10^−2^ M NaCl (∆); (**b**) EPS membrane with stirred solutions of C_f_ = 10^−3^ M NaCl (●), C_f_ = 10^−2^ M NaCl (o) and C_f_ = 10^−2^ M NaCl without solution stirring (x).

On the other hand, the influence of external factors (concentration level and solution stirring rate) on membrane potential values was considered for some membranes, and Figure 7 shows ΔΦ_mbr_ values of AMX and EPS membranes at two fixed NaCl concentration values (C_f_ = 10^−3^ M and 10^−2^ M), as well as the differences obtained based on the solution stirring condition (550 rpm or non-stirred for C_f_ = 10^−2^ M NaCl). These results show the total likeness of ΔΦ_mbr_ values of those corresponding to an almost ideal anion-exchange AMX membrane for the whole range of concentrations studied (even for C_v_ = 0.5 M NaCl), doing so independently of fixed concentration values due to the almost negligible contribution of the diffusion potential, though the EPS membrane shows a reduction in ΔΦ_mbr_ (absolute values) with the increase in the concentration of both stirred and non-stirred solution conditions.

## 5. Conclusions

Basic characteristics parameters of charged membranes, such as membrane fixed charge concentration (X_ef_) and permselectivity (anionic or cationic permselectivity), can be determined by analyzing membrane potential values using the Teorell–Meyer–Sievers model. This analysis also allows the estimation of both diffusion and Donnan (or interfacial) potential contributions, and since individual measurements of Donnan potential for solution(C_1_)//membrane//solution(C_2_) systems cannot be performed, this analysis provides information of great interest regarding charged membranes, as well as surface modification, by comparing original and modified membrane results. 

Both, X_ef_ and P(i) significantly depend on membrane material and structure, according to the results obtained regarding the different membranes studied. In particular, ion-exchange membranes (both positively and negatively charged) show very high counter-ion permeselectivity (≥90%), with almost total exclusion of co-ions and, consequently, high Donnan potential contributions for a wide range of NaCl solution concentrations due to the high X_ef_ values, as well as their denser structures and/or hydrophobic characters. However, electrolyte inclusion in the membrane structure associated with concentration gradient increase, with a shielding effect on the charged sites caused by the counter ions, seems to be the main reason for the lower permselectivity and higher diffusion potential contribution exhibited by hydrophilic membranes. Low pore-radii, rather than low porosity, seems to control the transport of ions through the nanoporous alumina membranes analyzed.

## Figures and Tables

**Table 1:** Effective concentrations of fixed charge in the membrane (X_ef_), anion transport number (t_−_), fit error and membrane anionic permselectivity (P(−)) for different positively charged membranes.

Membrane	X_ef_ (M)	t_−_	Error Fit (%)	P(−) (%)
AMX-Sb	+0.600	0.970	2.0	92.2
Ionics(+)	+0.220	0.960	2.8	89.6
Ionics(+)/H_2_SO_4_	+0.210	0.960	5.2	89.6
70CTA/30AlqCl	+0.034	0.914	4.4	78.0
Chitosan	+0.012	0.820	9.5	53.3
ALM-1/Al_2_O_3_	+0.040	0.860	2.2	63.6

**Table 2:** Effective concentrations of fixed charges in the membrane (X_ef_), cation transport number (t_+_), fit error and membrane cationic permselectivity (P(+)) for different positively charged membranes.

Membrane	X_ef_ (M)	t_+_	Error Fit (%)	P(+) (%)
Ionics(−)	−0.350	0.954	9.8	92.5
Ionics(−)/H_2_SO_4_	−0.300	0.932	10.2	88.9
Nafion-112	−0.235	0.906	5.3	84.1
Nafion-112/DTA^+^	−0.120	0.760	11.4	61.0
EPS	−0.180	0.868	7.5	78.5
RC–CE/AgNPs	−0.0184	0.702	9.6	51.5

## Data Availability

Not applicable.

## Supplementary Materials

The following are available online via the following link: https://www.mdpi.com/article/10.3390/membranes13080739/s1. Figure S1: Effect of concentration level, solution stirring and electrolyte on membrane potentials for the ALM-2 membrane; Figure S2: Salt permeability as a function of feed NaCl concentration for RC–CE and ALM-1 membranes; Figure S3. Salt diffusion as a function of feed NaCl concentration. (a) membrane RC-CE; (b) membrane ALM-1; Figure S4. SEM micrographs for membrane ALM/Al_2_O_3_: a) surface and b) cross-section; Figure S5: Strain-stress versus elongation for membranes: RC-CR (Δ) and RC-CE/AgNPs (▲).

## Nomenclature

a_j_	Solution activity
AgNPs	Silver nanoparticles
ALM	Nanoporous alumina membrane
AlqCl	AliquatCl (or C_25_H_55_N^+^Cl^−^)
C_v_	Solution concentration
$\bar{C_{j,i}}$	Ion concentration in the membrane
CTA	Cellulose triacetate
D_i_	Ion diffusion coefficient in the membrane
D_s_	Salt diffusion coefficient in the membrane
DTA^+^	n-dodecyltrimethylammonium cation
EIS	Electrochemical impedance spectroscopy
EPS	Microbial exopolysaccharide membrane
F	Faraday constant
I_i_	Current transport by ion i
I_T_	Total current transport for cations and anions
IS	Impedance spectroscopy
K_s,i_	Membrane partition coefficient
P(i)	Ionic permselectivity
PIM	Polymer inclusion membrane
R	Gas constant
RC	Regenerated cellulose
RC–CE	Regenerated cellulose membrane
r_p_	Membrane pore radii
T	Temperature of the system
t_i_	Ion transport number in the membrane
t^o^_i_	Ion transport number in solution
TMS	Teorell–Meyer–Siever
X_ef_	Effective membrane fixed charge concentration
z_i_	Ion valency
ΔΦ_mbr_	Membrane potential
ΔΦ_elect_	Electrode potential
ΔΦ_Don_	Donnan potential
ΔΦ_dif_	Diffusion potential in the membrane
ΔΦ°_dif_	Solution diffusion potential
ΔE	Measured potential
Θ	Membrane porosity

## References

[1] Mulder M. (1991). Basic Principles of Membrane Technology.

[2] Lakshminarayanaiah N. (1970). Transport Phenomena in Membranes.

[3] Helfferich F. (1962). Ion Exchange.

[4] Pourcelly G., Gavach C., Colomban P. (1992). Perfluorinated Membranes. Proton Conductors: Solids, Membranes and Gels: Materials and Devices.

[5] Zhang B., Fan L., Ambre R.B., Liu T., Meng Q., Timmer B.J.J. (2020). Advancing Proton Exchange Membrane Electrolyzers with Molecular Catalysts. Joule.

[6] Das G., Choi J.-H., Nguyen P.K.T., Kim D.-J., Yoon J.S. (2022). Anion Exchange Membranes for Fuel Cell Application: A Review. Polymers.

[7] Kotoka F., Merino-Garcia I., Velizarov S. (2020). Surface Modifications of Anion Exchange Membranes for an Improved Reverse Electrodialysis Process Performance: A Review. Membranes.

[8] Ahmad S., Nawaz T., Asghar A., Orhan M.F., Samreen A., Kannan A.M. (2022). An overview of proton exchange membranes for fuel cells: Materials and manufacturing. Int. J. Hydrog. Energy.

[9] Nebavskaya X., Sarapulova V., Butylskii D., Larchet C., Pismenskaya N. (2019). Electrochemical Properties of Homogeneous and Heterogeneous Anion Exchange Membranes Coated with Cation Exchange Polyelectrolyte. Membranes.

[10] Güell R., Anticó E., Kolev S.D., Benavente J., Salvadó V., Fontàs C. (2011). Development and characterization of polymer inclusion membranes for the separation and speciation of inorganic as species. J. Membr. Sci..

[11] Mareev S., Gorobchenko A., Ivanov D.A., Anokhin D., Nikonenko V. (2022). Ion and Water Transport in Ion-Exchange Membranes for Power Generation Systems: Guidelines for Modeling. Int. J. Mol. Sci..

[12] Kingsbury R., Wang J., Coronell O. (2020). Comparison of water and salt transport properties of ion exchange, reverse osmosis, and nanofiltration membranes for desalination and energy applications. J. Membr. Sci..

[13] Benavente J., Romero V., Vázquez M.I., Anticó E., Fontàs C. (2018). Electrochemical Characterization of a Polymer Inclusion Membrane Made of Cellulose Triacetate and Aliquat and Its Application to Sulfonamides separation. Separation.

[14] Kim M., Kim T. (2013). Integration of nanoporous membranes into microfluidic devices: Electrokinetic bio-sample pre-concentration. Analyst.

[15] Tang B., Bensdas S., Krajka V., May T., Moritz A., Constantinou I., Reichi S., Dietzel A. (2022). Self-loading microfluids platform with ultra-thin nanoporous membrane for organ-on-chip wafer-level processing. Front. Sens..

[16] Epsztein R., DuChanois R.M., Ritt C.L., Noy A., Elimelech M. (2020). Towards single-species selectivity of membranes with subnanometre pores. Nat. Nanotechnol..

[17] Sollner K. (1969). The electrochemistry of porous membranes, with particular reference to ion exchange membranes and their use in model studies of biophysical interest. J. Macromol. Sci..

[18] Boldini A., Porfiri M. (2022). A non-ideal solution theory for the mechanics and electrochemistry of charged membranes. Comput. Mater..

[19] Benavente J., Güell C., Ferrando M., López F. (2009). Electrical Characterization of Membranes. Monitoring and Visualizing Membrane-Based Processes.

[20] Kozmai A., Sarapulova V., Sharafan M., Melkonian K., Rusinova T., Kozmai Y., Pismenskaya N., Dammak L., Nikonenko V. (2021). Electrochemical Impedance Spectroscopy of Anion-Exchange Membrane AMX-Sb Fouled by Red Wine Components. Membranes.

[21] Szymczyk A., Dirir Y.I., Picot M., Nicolas I., Barrière F. (2013). Advanced electrokinetic characterization of composite porous membranes. J. Membr. Sci..

[22] Yaroshchuk A., Bruening M.L., Zholkovskiv E. (2019). Modelling nanofiltration of electrolyte solutions. Adv. Colloid Interface Sci..

[23] Ariza M.J., Benavente J. (2001). Streaming potential along the surface of polysulfone membranes: A comparative study between two different experimental systems and determination of electrokinetic and adsorption parameters. J. Membr. Sci..

[24] Escoda A., Lanteri Y., Fievet P., Déon S., Szymczyk A. (2010). Determining the dielectric constant inside pores of nanofiltration membranes from membrane potential measurements. Langmuir.

[25] Benavente J., Hilal N., Knayet M., Wright C.J. (2012). Use of Impedance Spectroscopy for Characterization of Modified Membranes in Membrane Modification: Technology and Applications.

[26] Macdonald J.R., Johnson W.B. (2018). Fundamentals of Impedance Spectroscopy in Impedance Spectroscopy: Theory, Experiment, and Applications.

[27] Ariza M.J., Cañas A., Benavente J. (2000). Electrical and surface chemical characterizations of the active layer of composite polyamide/polysulphone nanofiltration commercial membranes. Surf. Interface Anal..

[28] De Lara R., Benavente J. (2009). Use of hydrodynamic and electrical measurements to determine protein fouling mechanisms for microfiltration membranes with different structures and materials. Sep. Purif. Technol..

[29] González A.S., Vega V., Cuevas A.L., Martínez de Yuso M.V., Prida V.M., Benavente J. (2021). Surface Modification of Nanoporous Anodic Alumina during Self-Catalytic Atomic Layer Deposition of Silicon Dioxide from (3-Aminopropyl)Triethoxysilane. Materials.

[30] Peláez L., Romero V., Escalera S., Ibramova S., Stibius K., Benavente J., Hélix-Nielsen C. (2011). Electrochemical characterization of hydrogels for biomimetic applications. Polym. Adv. Technol..

[31] Benavente J., Muñoz A., Heredia A., Cañas A. (1999). Fixed charge and transport numbers in isolated pepper fruit cuticles from membrane potential measurements: Donnan and diffusion potential contributions. Colloids Surf..

[32] Teorell T. (1956). Transport phenomena in membranes. Discuss. Faraday Soc..

[33] Meyer K.H., Sievers J.F. (1936). La perméabilité des membranes I. Théorie de la perméabilité ionique. Helv. Chim. Acta.

[34] Acosta L.K., Law C.S., Santos A., Ferré-Borrull J., Marsal L.F. (2022). Tuning intrinsic photoluminescence from light-emitting multispectral nanoporous anodic alumina photonic crystals. APL Photonics.

[35] Kipke S., Schmid G. (2014). Nanoporous alumina membranes as diffusion controlling systems. Adv. Funct. Mater..

[36] Robinson R.A., Stokes R.H. (2012). Electrolyte Solutions.

[37] Masuda H., Fukuda K. (1995). Ordered Metal Nanohole Arrays Made by a Two-Step Replication of Honeycomb Structures of Anodic Alumina. Science.

[38] Romero V., Vega V., García J., Zierold R., Nielsch K., Prida V.M., Hernando B., Benavente J. (2013). Changes in morphology and ionic transport induced by ALD SiO_2_ coating of nanoporous alumina membranes. ACS Appl. Mater. Interfaces.

[39] Tavakolian M., Jafari S.M., van de Ven T.G.M. (2020). A review on surface-functionalized cellulosic nanostructures as biocompatible antibacterial materials. Nano-Micro Lett..

[40] Gotturk P.A., Sujanani R., Qian J., Wang Y., Katz L.E., Freeman B.D., Crumlin E.J. (2022). The Donnan potential revealed. Nat. Commun..

[41] Szymczyk A., Fievet P. (2006). Ion transport through nanofiltration membranes: The steric, electric and dielectric exclusion model. Desalination.

[42] Lanteri Y., Szymczyk A., Fievet P. (2008). Influence of Steric, Electric, and Dielectric Effects on Membrane Potential. Langmuir.

[43] Demish H.-U., Pusch W. (1979). Electric and electrokinetic transport properties of homogeneous weak ion exchange membranes. J. Colloid Interface Sci..

[44] Filippov A.N., Starov V.M., Konomemko N.A., Berezina N.P. (2008). Asymmetry of diffusion permeability of bilayers membranes. Adv. Colloids Interface Sci..

[45] Kimura Y., Lim H.J., Iijima T. (1984). Membrane potentials of charged cellulosic membranes. J. Membr. Sci..

[46] Nieto Castillo A., García-Delgado R.A., Cala Rivero V. (2012). Electrokinetic treatment of soils contaminated by tannery waste. Electrochim. Acta.

[47] George S.M. (2010). Atomic layer deposition: An overview. Chem. Rev..

[48] De Yuso M.D.V.M., Cuberes M.T., Romero V., Neves L., Coelhoso I., Crespo J.G., Rodríguez-Castellón E., Benavente J. (2014). Modification of a Nafion Membrane by N-Dodecyltrimethylammonium Cation Inclusion for Potential Application in DMFC. Int. J. Hydrog. Energy.

[49] Ferreira A.R.V., Torres C.A.V., Freitas F., Sevrin C., Gandis C., Reiss M.A.M., Alves V.D., Coelhoso I.M. (2011). Characterization of biodegradable films from the extracellular polysaccharide produced by *Pseudomonas oleovorans* grown on glycerol byproduct. Carbohydr. Polym..

[50] Ferreira A.R.V., Torres C.A.V., Freitas F., Sevrin C., Gandis C., Reiss M.A.M., Alves V.D., Coelhoso I.M. (2016). Development and characterization of bilayer films of FucoPol and chitosan. Carbohydr. Polym..

[51] Benvente J., García M.E., Urbano N., López-Romero J.M., Contreras-Cáceres R.C., Casado-Rodríguez M.A., Hierrezuelo J. (2017). Inclusion of silver nanoparticles for improving regenerated cellulose membrane performance and reduction of biofouling. Int. J. Biol. Macromol..

[52] Kum S., Lawler D.F., Katz L.E. (2020). Separation characteristics of cations and natural organic matter in electrodialysis. Sep. Purif. Technol..

[53] Park J.-S., Shin M.S., Sekhon S.S., Choi Y.-W., Yang T.-H. (2011). Effect of annealing of Nafion recast membranes containing ionic liquids. J. Korean Electrochem. Soc..

[54] Zhu L.Y., Li Y.C., Liu J., He J., Wang L.Y., Lei J.D. (2022). Recent Developments in High-Performance Nafion Membranes for Hydrogen Fuel Cells Applications. Pet. Sci..

[55] Yan N., Sujanani R., Kamcev J., Galizia M., Jang E.-S., Paul D.R., Freeman B.D. (2022). Influence of fixed charge concentration and water uptake on ion sorption in AMPS/PEGDA membranes. J. Membr. Sci..

