# Supplementary material for: Electrochemical Characterization of Charged Membranes from Different Materials and Structures via Membrane Potential Analysis
Source: Membranes (Basel). 2023 Aug 17;13(8):739. doi: 10.3390/membranes13080739 (PMC10456455; doi:10.3390/membranes13080739)
Supplement: Supplementary file 1 [file membranes-13-00739-s001.zip › membranes-2485951-supplementary.pdf]

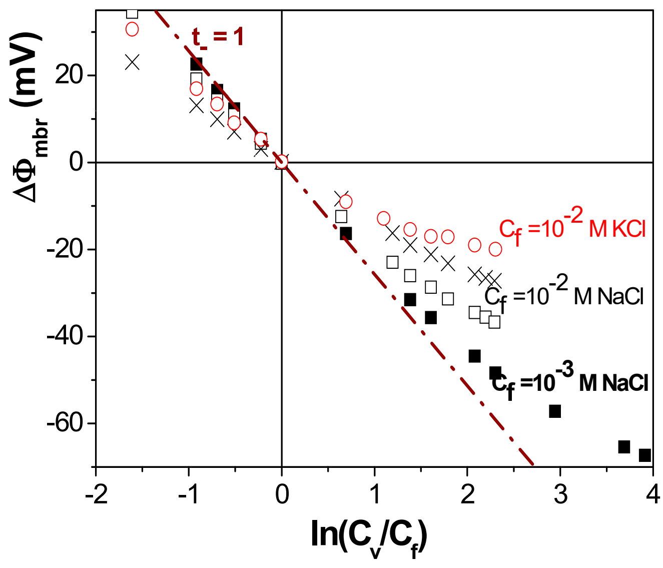

Figure S1. Membrane potential as a function of solution concentration ratio for nanoporous alumina membrane ALM-2. C_f = 0.01 M NaCl: solutions stirred at 550 rpm (□) and non-stirred solutions (x); stirred solutions: $C_f = 10^{-3}$ M NaCl (■) and $C_f = 10^{-2}$ M KCl (o). Theoretical values for an ideal cation-exchanger (dashed-dot line).

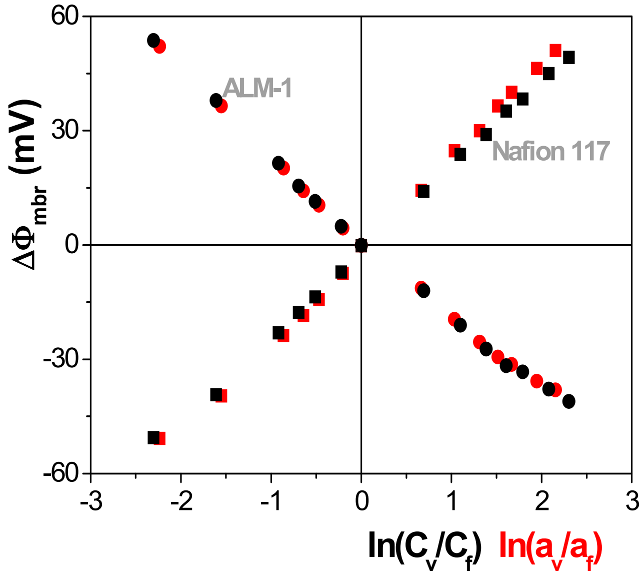

Figure S2. Membrane potentials as a function of: ln(C_f/C_v) for Nafion-117 membrane (■) and ALM-1 membrane (♦); ln(a_f/a_v) for Nafion-117 membrane (■) and ALM-1 membrane (♦).

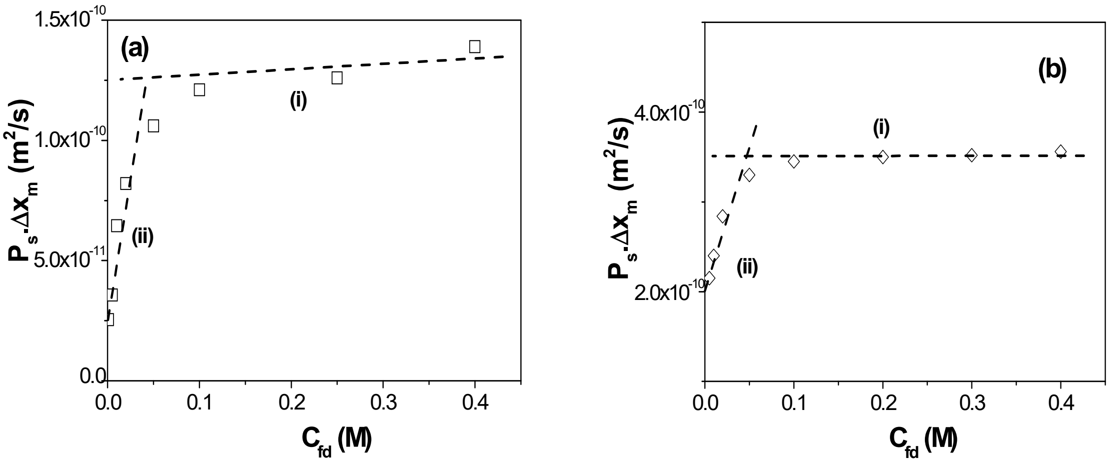

Figure S3. Salt diffusion as a function of feed NaCl concentration. (a) membrane RC-CE; (b) membrane ALM-1.

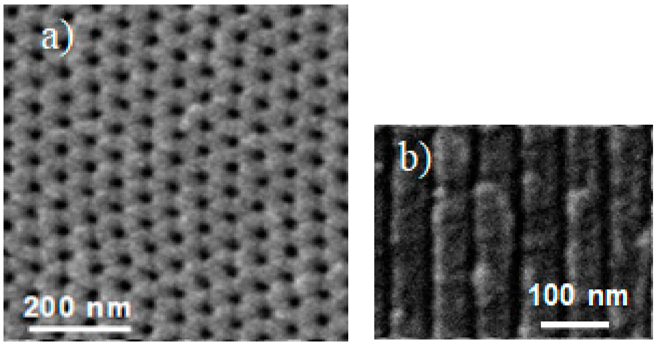

Figure S4. SEM micrographs for membrane ALM/Al_2O_3: a) surface and b) cross-section.

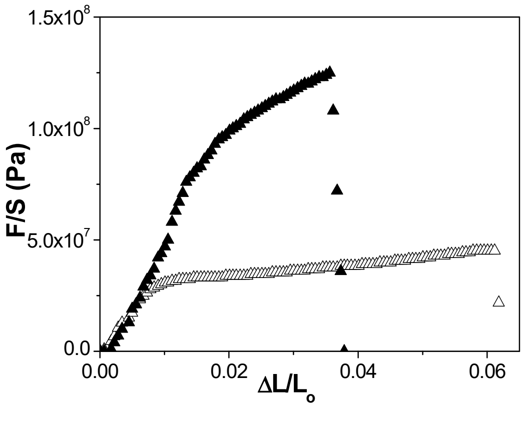

Figure S5: Strain-stress versus elongation for membranes: RC-CR (Δ) and RC-CE/AgNPs (▲).
